# A Successful Method of Treating Acute Rheumatism

**Published:** 1855-06

**Authors:** A. B. Garrod

**Affiliations:** Physician to University College Hospital


A Successful Method of Treating Acute Rheumatism. By A. B. Garrod, M. D., Physician to University College Hospital. —The author after a few preliminary remarks, observed, that he was induced, in May, 1852, to try a new method of treating acute rheumatism; and finding great success at first, resolved steadily to pursue the plan, and has done so up to the present time. The object of his communication has been to record the method adopted by him, and also the results obtained in fifty-one cases of rheumatic fever which have been admitted, under his care, in University College Hospital, during the last two years and three quarters. The main part of his plan of treatment consists in the administration, in a diluted form, of two scruple doses of bicarbonate of potash, every two hours, day and night, until the patient has been free from all articular affection and febrile disturbance for two or three days, using local depletion over the heart's region, if any cardiac disease is present or threatened. The author then detailed three cases of rheumatic fever, illustrating this mode of treatment; the first, a girl, ten years old, in which the duration under treatment was five days, the total duration eight; the second, a young man, aged twenty, with a complication of heart disease, where the duration under treatment was eight; the total duration fifteen days; the third, a young woman, aged eighteen years, in the fifth attack, the former ones having always lasted for a month or five weeks, but which, by the adoption of this plan, yielded in nine days, total duration being but thirteen days, four having elapsed before her admission into the hospital. He afterwards gave a table of fifty-one cases of acute rheumatism; and of each patient the following particulars are noted:—The age, occupation, hereditary predisposition; the number and causes of attack; the symptoms before admission; the symptoms during treatment; the nature of treatment; and the duration of the disease. From these cases, the following deductions are made, viz: that in twenty males the duration of the disease under treatment averaged between six and seven days, and the total duration between eleven and twelve days; and in thirty-one females, the disease under treatment averaged from seven to eight days, and the total duration between fifteen and sixteen days—giving in all an average under treatment of seven days and a half; and, for the total duration, about thirteen days and a half. The author then alluded to the influence of the bicarbonate of potash when administered in large and frequent doses upon the different organs and functions of the body; and remarked that it produces neither nausea, vomiting, nor purging—in fact, no symptom of gastro-intestinal irritation. It now induces a strongly alkaline condition of the urine, causes it to effervesce freely, with excess of acid, but does not appear to promote an increase in the quantity of the secretion. It appears to render the secretion of the skin less acid—sometimes almost neutral. That it acts as a powerful controller of the heart's action, reducing greatly the frequency of the pulse, but without causing the faintness often produced by digitalis, colchicum, &c. That it probably increases the alkalinity of the serum of the blood, and diminishes the coagulability of the altered fibrine occurring in rheumatic fever; and hence, probably, checking or preventing the deposits of lymph on the endo- or peri-cardium. He (Dr. Garrod) stated his opinion, that the influence of the bicarbonate was felt not only in shortening the duration of the articular affection, but also in preventing or moderating the cardic disease. After enumerating many details of the method adopted, and the value of certain adjuncts, as opium, calomel, and occasional general depletion, he proceeded to recommend a plan of treatment, which, from his experience, he considered calculated to ensure the greatest amount of success, and thought it probable that the total duration of the disease might, on the average, be reduced to about ten days,—provided that the treatment was adopted early, and no serious complication existed.

Dr. COPLAND inquired whether the author of the paper had used the bicarbonate of soda, and what were the results. The alkaline treatment of rheumatism had been long in use. In his treatise on that disease he had recommended, after the proper evacuation of the bowels, that the decoction of bark with alkalies should be resorted to. He mentioned some cases in which the disease appeared to have been cut short by the following plan of treatment:—A powder, consisting of half a drachm of magnesia and the same quantity of milk of sulphur was given night and morning, and bark with alkalies, as mentioned above, three times during the day. Alkalies had been recommended many years since by a friend of his, in a work entitled, "The Simple Treatment of Disease."

Dr. Dickson remarked that Dr. Garrod's cases occurred in hospital patients, and we were ignorant of the real results of the treatment recommended. He could not but think that such large doses of alkalies must in the end be very injurious. He thought the treatment of rheumatism should be of a more vigorous character, and that mercury and other agents which had a decided effect on the liver would relieve the patient quicker than any other remedies. He had seen much benefit result in such cases from quarter of a grain doses of alcoholic extract of aconitine administered every six hours. He should be glad to know the ultimate effect of such large doses of alkalies on the system.

Dr. Copland had seen good effects from the alcoholic extract of aconitine. In some cases of protracted rheumatism, anæmia presented itself, and was best treated by iron and bark.

Dr. Garrod had not tried the bicarbonate of soda, his object being to determine the effects of simple treatment, and the results of a single remedy. His experience of the benefit of the potash treatment had been confirmed by that of many of his friends. He did not mean to say that no other remedies should be employed; on the contrary, opiates, or extract of aconitine, might be often used with great advantage. Bicarbonate of soda would probably be as useful as the potash; but he had not had time to determine this. Several of the cases he had recorded had been cured in a very short time. He would answer, that in no case had any injurious effects to the system followed the use of the potash, as he had seen patients at the end of two or three years after an attack, and these had never had rheumatism after. The cases he had recorded were not all hospital patients, several of them having occurred in private practice. He did not think rheumatism produced anæmia; but it might occasionally require iron and quinine for its cure. The late Dr. Taylor had employed aconitine, but with little success.

Dr. Webster, after speaking of the great diversity of opinion which existed with respect to the treatment of rheumatism, said that he felt inclined to doubt the efficacy of a new remedy, so many having been vaunted, and afterwards abandoned. It must be remembered, that Dr. Garrod's cases occurred in patients whose ages varied from ten to twenty; and in these the alkaline treatment might be of use. But he (Dr. Webster) should hesitate to employ it in old people. In some cases Dr. Garrod had employed leeches and opium, and no doubt these means had had an effect on the result. Rheumatism, a few years since, was treated almost exclusively with acids. For his own part, he should, in young subjects, trust rather to calomel and opium than to alkalies.

Dr. O'Connor remarked, that lemon-juice had been generally abandoned in hospital practice from its producing very great prostration. He had used bicarbonate of potash in six cases of rheumatism, one being a weakly child, seven years of age, with benefit. He had trusted exclusively to this remedy—the only addition to it being the envelopment of the affected limb in flannel, sprinkled over with sulphur. He had, in cases attended with great prostration, used, with benefit, Bastick's cod-liver oil and quina. Dr. Budd was in the habit of using the nitrate and carbonate of potash, but in smaller doses than those recommended by Dr. Garrod. Dr. Corrigan had treated the disease exclusively with opium; Dr. Graves with opium and calomel; and Dr. Basham with large doses of the nitrate of potash.

Mr. Streeter said that it was astonishing to observe how remedies for rheumatism rose and sunk, and that we kept returning to old forms of treatment with some modifications. Nitrate of potash, tartar emetic, &c., were useful in the early stage of acute rheumatism; and when the inflammatory symptoms were gone, potash and bark were of service. Dr. Haygarth had recommended bark eighty years ago. Dr. Garrod's cases had occurred in years when the constitution could not bear depletion. There was something in the state of the system of late years which seemed altogether to prohibit blood-letting. Mr. Streeter then made some remarks on the importance of care as to diet after a rheumatic attack, particularly as regarded an excess of animal food, from which a relapse might be feared.

Dr. Radcliffe remarked, that to determine with precision the value of any plan of treating rheumatism, it was essential to know the natural duration of the disease. He believed that in one of the Brussels Hospitals it had been shown that the average duration was ten or twelve days.

Dr. Rogers had used the lemon-juice in cases of rheumatism, and had never observed any depressing effects from its use; even in cases where rheumatism supervened on phthisis, he had found it of the greatest service. Treatment by lemon-juice was, in fact, the alkaline treatment. Lemon-juice was an acid citrate of potash; the alkali was set free in the stomach, and eliminated the materia morbi from the system.

Dr. Basham remarked, that the discrepancies with respect to the treatment of rheumatism originated in the confounding of various forms of the disease together. The acute form of the affection was a blood disease, the fibrinous portion of the fluid being in excess, whilst the salts were deficient in quantity. He had employed the nitrate of potash on the plan recommended by Gendrin and Martin Solon, with much success; but this plan was not new. Dr. Brockelsby, at the close of the last century, used this medicine in doses of two to four drachms. He (Dr. Basham) believed that any treatment based on the principle of eliminating the materia morbi from the blood was the best. He had seen much relief to the pain in the joints follow the local application of spongio-piline saturated with nitre.

Dr. GARROD said, in reply to Dr. Webster's objection, that his patients were all young, that acute rheumatism was a rare disease after the age of forty-five.—*Lancet.*

